# Fano Resonance on Nanostructured Lithium Niobate for Highly Efficient and Tunable Second Harmonic Generation

**DOI:** 10.3390/nano9010069

**Published:** 2019-01-05

**Authors:** Zhijin Huang, Huihui Lu, Hanqing Xiong, Yang Li, Huajiang Chen, Wentao Qiu, Heyuan Guan, Jiangli Dong, Wenguo Zhu, Jianhui Yu, Yunhan Luo, Jun Zhang, Zhe Chen

**Affiliations:** 1 Guangdong Provincial Key Laboratory of Optical Fiber Sensing and Communications, JinanUniversity, Guangzhou 510632, China; huangzhijin@foxmail.com (Z.H.); liyangjnu@gmail.com (Y.L.); chj2018522@126.com (H.C.); jldong@jnu.edu.cn (J.D.); ccdbys@163.com (J.Z.); 2 Key Laboratory of Optoelectronic Information and Sensing Technologies of Guangdong Higher Education Institutes, Jinan University, Guangzhou 510632, China; thuihuilu@jnu.edu.cn (H.L.); hanqingx1203@gmail.com (H.X.); zhuwg88@163.com (W.Z.); kensomyu@163.com (J.Y.); yunhanluo@163.com (Y.L.); thzhechen@163.com (Z.C.)

**Keywords:** lithium niobate, harmonic generation and mixing, Fano resonance

## Abstract

Second harmonic generation (SHG) is an important nonlinear process which is critical for applications, such as optical integrated circuit, nonlinear microscopy, laser, etc. Many challenges remain in the improvement of nonlinear conversion efficiency, since the typical value is of only 10^−5^ in nanostructures. Here, we theoretically demonstrate a periodic structure consisting of a lithium niobate (LN) bar and an LN disk, on a nanoscale (~300 nm) thin-film platform, which is proposed for a highly efficient SHG. By breaking the structure symmetry, a Fano resonance with a high Q, up to 2350 and a strong optical field enhancement reaching forty-two folds is achieved, which yields a high conversion efficiency, up to 3.165 × 10^−4^. In addition to its strong second harmonic (SH) signal, we also demonstrate that by applying only 0.444 V on the planar electrode configurations of the nanostructured LN, the wavelength of SH can be tuned within a 1 nm range, while keeping its relatively high conversion efficiency. The proposed structure with the high nonlinear conversion efficiency can be potentially applied for a single-molecule fluorescence imaging, high-resolution nonlinear microscopy and active compact optical device.

## 1. Introduction

Second Harmonic Generation (SHG) is a nonlinear optical process of converting two photons into one, while doubling its frequency [1,2]. It is a process that results from the interaction of an incident wave at the fundamental wavelength (FW), with material that has a second-order nonlinearity. It has significant applications in domains such as quantum communication light sources at exotic wavelength [3], high-resolution nonlinear optical microscopy [4], morphology detection of metallic nano-objects [5], medical imaging [6], etc. Structures enabling a high light confinement, such as plasmonic nanostructures [7] and resonantly-enhanced nano-patterned metasurface arrays, are proposed, in order to enhance the second harmonic (SH) conversion efficiency. For example, by placing a nonlinear ZnO nanowire into a plasmonic hot-spot, created by a gold pentamer oligomer, a high SH conversion efficiency of 3 × 10^−5^ is achieved [8]. Similarly, benefiting from the local field intensification by plasmonic nanocavity and by reflection from the Fabry-Perot cavity, Liu et al. demonstrated an efficient SHG nanostructure by a single CdS nanobelt, deposited onto the SiO_2_/Au substrate [9]. Another paradigm of boosting the SH signal is to break the structure symmetry, in order to create a Mie type localized modes with high quality factor Fano resonances in GaAs metasurface, which has been demonstrated in Reference [10], where a high SH conversion efficiency of 6 × 10^−5^ is achieved.

Even though the nano-patterned structure helps to greatly enhance the SHG, the state-of-the-art configuration shows that most of the reported SHG conversion efficiency are around the scale of 10^−5^, which is still low for applications such as light-emitting nano-probes for single-molecule fluorescence imaging [11,12,13,14]. This insufficient conversion efficiency might partly be due to the employment of metallic materials with a dissipative loss in the plasmonic resonance. On the other hand, the utilization of materials, such as GaAs, might suffer from a high-pump influence inducing irreversible damage, due to the thermal heating caused by a two-photon absorption [15].

As for the choice of material, lithium niobate (LN) is a biocompatible material which has a large second-order nonlinearity (the largest one is *d_33_* = 27 pm/V at 1550 nm) [16,17,18]. Even though it shows slightly weaker nonlinear properties than some semiconductor materials, it has a wide transparency spectral range spanning visible to infrared light (0.42–5 μm). Another interesting property of LN is the linear electro-optic (EO) effect, where the largest EO coefficient *r_33_* is 30.8 pm/V [1,19]. Combining the EO effect and the second-order nonlinearity, one can build a tunable SHG nanostructure carved onto LN. In addition, the commercially available, nanometrical, thin, crystalline LN on insulator, offer chances to design structures with high field localizations [20,21,22,23,24,25,26]. In terms of obtaining a high field confinement, Fano resonance is an excellent choice. This type of resonance arises from the interference of the bright super-radiant and the dark sub-radiant modes in nanostructures, which has found a myriad of applications in physical, chemical and quantum information, biological sciences, etc. [27]. The asymmetric line shape allows to have high *Q* factor and a strong light confinement resonance, which is beneficial for boosting the nonlinear conversion. 

**Figure 1:**
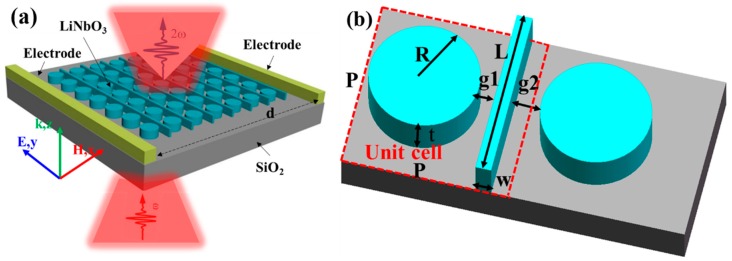
(**a**) Schematic of the TFLN periodic structure configuration for the Second Harmonic Generation (SHG). The distance of the electrodes is *d*. (**b**) Dashed square is one of the basic ‘meta atom’. The pitch of the meta-atom is *P*, the radius of the lithium niobate (LN) disk is *R*, and the thickness of the TFLN is *t*. The width and length of the LN bar is *w* and *L*, respectively.

In this paper, we theoretically present a tunable SHG via Fano resonances, obtained on an X-cut LN periodic structure, which consists of LN disks and LN bars (schematically shown in Figure 1a). The resonance *Q* factor can yield high up to ~2350 and the optical field enhancement factor *f_opt_* yield up to forty-two folds at the resonant wavelength. The SHG conversion efficiency is predicted to be as high as 3.165 × 10^−4^, due to the strong field localization in all the dielectric LN periodic structure of the Fano resonance structure. In addition, the tunability of the SH wavelength via the EO effect, with an external voltage as small as 0.444 V, has been demonstrated. First, we start the linear simulation, which does not take into account whether the second-order nonlinearity of the material is carried out, in order to optimize the structure parameters. Second, simulations which take the nonlinearity into account are presented. The SH enhancement and SHG conversion efficiency of the optimized structure is quantified. Next, how the SHG conversion efficiency varies with respect to the EO effect tuned refractive index variation, is also investigated. In the end, the state-of-the-art SHG configurations are compared and some perspectives are provided.

## 2. Design, Results, and Discussions

### 2.1. Linear Simulation 

In nanostructure configurations, phase matching is negligible, since the thickness is usually smaller than a wavelength [28]. Other factors, such as field localization and structure symmetry play important roles in boosting an efficient nonlinear conversion. A systematic 3D Finite-Difference Time-Domain (FDTD) (Lumerical FDTD Solution) parametrical study, without taking the nonlinearity into account, was performed, to achieve a high-Q resonance and strong light confinement at the pump light wavelength. The study was conducted by optimizing the structure parameters, such as the length of the LN bar *L*, the thickness of the thin film lithium niobate (TFLN) *t*, and the degree of the asymmetric distance of the bar from the disks Δ*g = g*1 − *g*2. One-unit cell or ‘meta atom’ of the structure (shown as the dashed square in Figure 1b) was considered in the 3D-FDTD calculations. It consisted of a computational window of 1100 nm × 1100 nm in the x-y plane. The structure was illuminated by a normally incident y-polarized pulsed plane wave from the silica substrate. Bloch periodic boundary condition was applied in the x and y directions. PML (perfectly matched layer) was applied along the z direction. The transmission spectra was obtained by performing the Fourier transform of the time varying electric fields on the detector plane, which was placed four micrometers above the top surface of the nano structure, and was normalized with the incident light. A nonuniform grid resolution with a minimum of 10 nm was applied in the x, y, and z direction. The refractive index of the silica was set to 1.45 (at the wavelength of 1550 nm). The anisotropic and dispersive nature of the LN was taken into account by describing LN’s refractive index, according to the Sellmeier Equations [29], as follows:

(1) $$n_{e}=1+\frac{2.9804\lambda^{2}}{\lambda^{2}-0.02047}+\frac{0.5981\lambda^{2}}{\lambda^{2}-0.0666}+\frac{8.9543\lambda^{2}}{\lambda^{2}-416.08}$$

(2) $$n_{o}=1+\frac{2.6734\lambda^{2}}{\lambda^{2}-0.01764}+\frac{1.2290\lambda^{2}}{\lambda^{2}-0.05914}+\frac{12.614\lambda^{2}}{\lambda^{2}-474.6}$$

**Figure 2:**
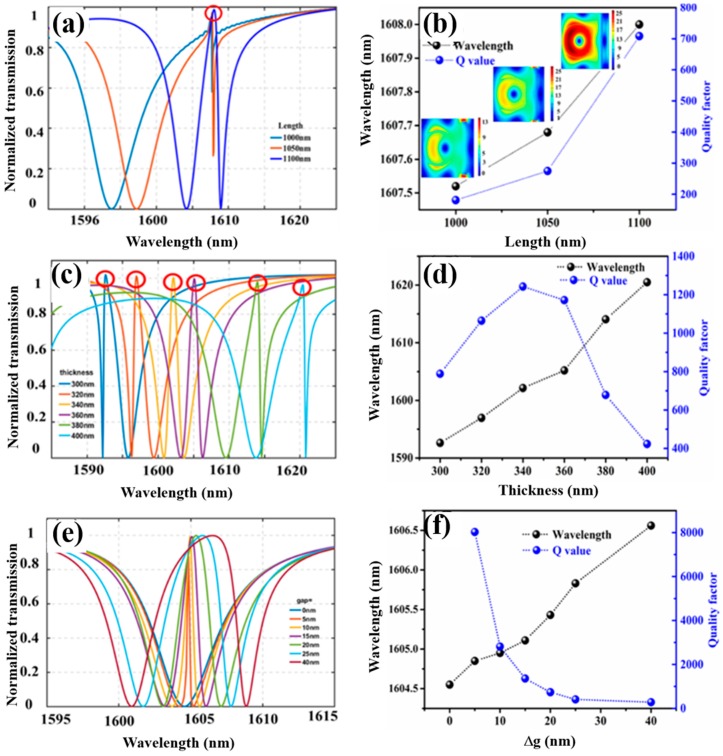
Normalized transmission of varying (**a**) the length of the LN bar *L*, while other parameters are fixed, with *t* = 360 nm, Δ*g* = 20 nm, *R* = 350 nm, and *w* = 100 nm, (**c**) the TFLN thickness *t*, while other parameters are fixed, with *L* = 1100 nm, Δ*g* = 20 nm, *R* = 350 nm, and w = 100 nm, (**e**) the asymmetric distance between the bar edges to the edges of the neighboring LN disks Δ*g = g*1 *− g*2, while the other parameters are fixed, with *L* = 1100 nm, *t* = 340 nm, *R* = 350 nm, and *w* = 100 nm. The peak marked by the red circles are considered. Fano resonance peak wavelength and the corresponding resonance *Q* factor of varying, (**b**) the length of the LN bar (the insets show the field distribution along the middle of the disk), (**d**) the TFLN thickness, (**f**) the asymmetric distance between the bar edges to the edges of the neighboring LN disks Δ*g = g*1 − *g*2.

Simulation results are shown in Figure 2. Figure 2a shows the various transmission spectra with respect to different LN bar length *L* while Figure 2b is the Fano-resonant wavelength and the Q factor (as defined by λ_0_/Δλ, where λ_0_ is the resonant wavelength and Δλ is the full-width at a half minimum (FWHM) of the resonance), corresponding to the structure length of the different LN bar in Figure 2a. Note that the parameter of Δ*g* was set to be 10 nm (corresponding to the asymmetric distance between the LN bar and the LN disks), since breaking the structure symmetry was needed to boost the nonlinear conversion efficiency, and this was further substantiated by the simulation results of the varying Δ*g* in Figure 2e.

As we can see from Figure 2a, at the wavelength of around 1607 nm, there is an asymmetric Fano sharp peak between the two broad transmission dips (highlighted as red circles). This peak is chosen as the pump wavelength for the exploitation of the SHG, since it corresponds to a high-Q dielectric mode with high transmission characteristics. Figure 2b shows that the variation of the LN bar length *L* has a little effect on the resonance peak wavelength, whereas the resonance *Q* is greatly affected. The longer the LN bar length *L*, higher the resonance *Q* achieved. The mode field distributions corresponding to the different *L* are shown in the inset of Figure 2b. As the LN bar length increases up till an LN nanobeam, i.e., *L* equal the period of the meta-atom of 1100 nm, a high *Q* of ~700 and a dielectric mode is achieved. Note that, other ways of breaking the symmetry of the structure, such as creating a notch in a cubic, can couple to an air mode and a high-Q Fano resonance, but it is not suitable for the SHG. Figure 2c shows the transmission curves under the different TFLN thickness *t*, while Figure 2d is the corresponding resonance wavelength and the Q factor. Since the thickness of the TFLN greatly affects the resonance condition, the resonance wavelength, therefore, is varied, as shown in the dashed curve with black olives in Figure 2d. As to how the resonance *Q* factor varies with respect to *t*, the resonance FWHM become narrower at the vicinity of *t* = 340 nm (orange curve in Figure 2c and dashed curve with blue olives in Figure 2d). Consequently, the thickness *t* is set to 340 nm by the criteria of choosing a high *Q* factor resonance. In addition, such thickness is commercially available.

Figure 2e shows the transmission curves under different Δ*g*, while Figure 2f is the corresponding resonance wavelength and the *Q* factor. The LN bar here is to aid the excitation of modes that can confine energy in the nonlinear material LN. However, as we can see from the blue curves in Figure 2e, corresponding to the Δ*g* = 0, there is no Fano-resonance peak in the transmission. This is because in the case of Δ*g* = 0, the distance of the LN bar to the two neighboring LN disks are the same. The symmetrically distributed LN disks, with respect to the LN bar, leads to the coupling of modes of different polarity. Therefore, causing an almost total destructive interference of the modes between the two neighboring disks. Upon creating broken-symmetry of the structure, a dielectric mode, where the light is confined inside the LN disk, is achieved. It manifests as a Fano peak in the transmission curves in Figure 2e. The parameter Δ*g* mainly affects the coupling efficiency of the mode and the resonance *Q*, as indicated by the dashed blue line with blue olives, in Figure 2f. Whereas, the resonance wavelength shown as black dashed curve with black olives in Figure 2f is slightly affected. Although, smaller the Δ*g* is, the higher the resonance *Q* is, in the periodic structure case. A higher *Q* resonance is more difficult to excite, since it allows a little fraction of light to be coupled inside the cavity. As a consequence, the extinction ratio of the transmission is degraded [30,31]. Consequently, the Δ*g* is chosen to be 10 nm by making a compromise between *Q* and the mode excitation efficiency.

**Figure 3:**
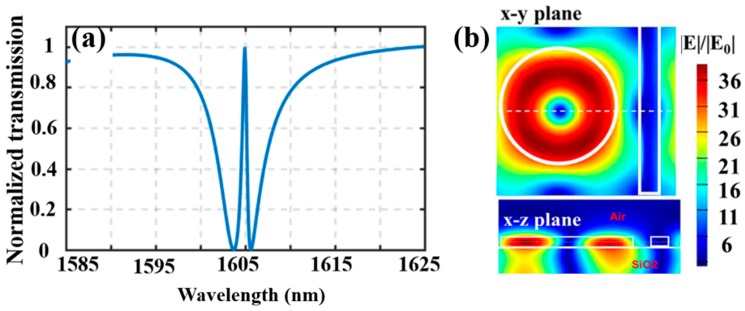
(**a**) Transmission spectrum corresponds the structure parameters of *R* = 350 nm, *L* = 1100 nm, *t* = 340 nm, *w* = 100 nm, *g*1 *=* 155 nm, *g*2 *=* 145 nm, and Δ*g* = 10 nm. (**b**) Electric field distribution of the x-y plane cross-section (cut through the middle of the TFLN), where its amplitude is divided with respect to the incident electric field strength E_0_. The bottom is the electric field distribution in the x-z plane cross-section (cut through the middle of the LN disk, as shown as the dashed line through the disk), where its amplitude is divided with respect to the incident electric field strength E_0_.

According to the above parametrical study, the optimized parameters of the proposed periodic structure are as follows: *R* = 350 nm, *L* = 1100 nm, *t* = 340 nm, *w* = 100 nm, *g*1 *=* 155 nm, *g*2 *=* 145 nm, and Δ*g* = 10 nm. With these parameters, the obtained transmission spectrum is shown in Figure 3a, where a Fano resonance with a *Q* of 2350 and an almost 100% to 0% ER, at the wavelength of 1605 nm, is achieved. A continuous wave simulation at the resonance peak wavelength is carried out and the electric field distribution (divided by the incident averaged electric field strength *E_0_*), for different cross-sections are shown in Figure 3b. As we can see from these field distributions that most of the energy is confined in the LN disk region and there is a nodal point in the center of the disk. The localized electric field yields about thirty-six times larger than the incident field in amplitude. In order to quantify this light localization effect, an enhancement factor *f_opt_*, which is defined as the ratio of the volumetric electric field amplitude integration in the pattern LN region, with respect to the same quality in the unpatterned LN region, is employed [20]. It denotes as follows:

(3) $$f_{opt}=\frac{{∭}_{\mathrm{structured}}\left|E\left(x,y,z\right)\right|dxdydz}{{∭}_{\mathrm{unstructured}}\left|E\left(x,y,z\right)\right|dxdydz}$$

An optical field enhancement factor *f_opt_* of up to ~42.42 is achieved with the optimized structure parameters, which is promising for nonlinear applications.

### 2.2. Nonlinear Simulation

In this section, the geometric parameters determined by the above linear simulations are employed, in order to investigate its nonlinear effects. In order to efficiently enhance the second-order nonlinearity, we orient the largest electric field component of the Fano resonance mode (x-direction as shown in Figure 1a) along the crystalline direction, where lies the largest second-order nonlinear coefficient. Consequently, an x-cut thin-film lithium niobate is chosen, which is favorable for the electro-optic tunability and its coplanar electrodes fabrication. As a result, the considered nonlinear susceptibility of the LN were *χ_x_*_x_ = 66 pm/V, *χ_yy_* = 0 pm/V, *χ_zz_* = 6 pm/V [1,32]. Whereas, the second-order nonlinearity of the SiO_2_ was neglected, since it is much smaller than that of LN and the light is confined mostly in the LN region. The calculation of nonlinear polarization is incorporated in the 3D-FDTD algorithm. The calculation windows, meshing size and the boundary conditions were the same as that employed for the linear simulations.

**Figure 4:**
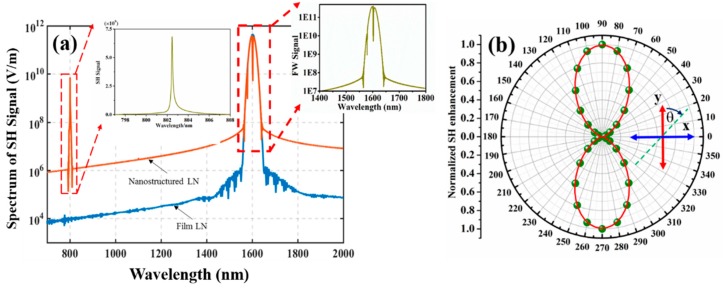
(**a**) The simulated SH signal with the optimized structure parameters. The orange curve is obtained for the nanostructured LN while the blue curve is obtained for the unstructured TFLN. The inset shows a magnified view of the SHG spectrum which peaks at 802 nm. (**b**) Polar diagram of the SH signal (normalized to the maximum) for the nanostructured LN, under different incident polarization angles. The polarization angle *θ* is defined as shown in the inset.

The structure is illuminated by a normal-incident narrow-band Gaussian pulse with a FWHM of 15 nm and a field amplitude of 1.55 × 10^8^ V/m at 1605 nm, corresponding to a peak-field intensity of 3.2 GW/cm^2^. The transmission spectrum was calculated via Fourier transform of the time varying fields at the detector plane, which was several micron meters above the structure and the results are shown in Figure 4a. Orange curve in Figure 4a corresponds to the transmittance of the nanostructured LN, while the blue curve in Figure 4a corresponds to the transmittance of the unstructured thin LN. As we can see from Figure 4a, at the vicinity of half the fundamental wavelength of 1605 nm, there was a strong signal for the nanostructured LN. In order to quantify the SH enhancement, a factor *τ* was employed and it was defined as the ratio of the SH intensity in the nanostructured LN, with respect to the same quantity in the unstructured thin LN, as follows [33]:

(4) $$\tau =I_{\mathrm{structured}-\mathrm{LN}}/I_{\mathrm{unstructured}-\mathrm{LN}}$$

By this quantification, a large enhancement factor *τ* of up to 10^11^ was achieved for our proposed TFLN periodic structure with Fano resonance. Even though the result was obtained in a periodic structure which might be degraded in the real fabricated customized structure size, it showed the potential of a great enhancement of the SH signal, thanks to the high in-plane electric field confinement in the nanostructured LN. Another quantification of the SH signal was the SHG conversion efficiency which was defined as [34]:

(5) $$\eta_{SH}=P_{SH}/P_{FF}$$

where $P_{FF}=I_{FF}\cdot A$ represents the power at the fundamental wavelength, $I_{FF}$ is the pump light intensity, and *A* is the area of a unit cell of the nanostructured LN. The conversion efficiency of the nanostructured LN was estimated to be as high as 3.165 × 10^−4^.

In order to investigate the influence of the incident polarization on the SH signal, a polarimetric diagram of the normalized SH intensity in the nanostructured lithium niobate versus the incident light angle is shown in Figure 4b. As expected, the largest SH intensity was achieved when the incident light oriented along the y-direction, which was defined as polarization angle *θ* = 0°, as shown in Figure 4b. The intensity of the SH signal gradually decreased as the polarization direction of the incident light pointed away from the y-direction. Finally, the SH intensity became close to zero, as the polarization direction of the incident light pointed along the x-direction, i.e., polarization angle *θ* = 90°.

Table 1 shows the performance comparison of the different structure configuration for SHG, in the literature. The all dielectric nanostructured LN proposed here, achieved a high SH conversion efficiency up to the order of 10^−4^, whereas, other configurations, with material such as semiconductors and metallic structure usually had a conversion efficiency not exceeding 10^−5^. In terms of the large SH enhancement factor *τ*, the quantification of the proposed structure here corresponded to a periodic structure, it therefore, exceeded several orders larger than those in the fabricated structure configurations. Overall, the all dielectric Fano-resonance-based LN proposed here, showed a better performance in terms of the SH enhancement and yielded a higher SH conversion efficiency than that of the plasmonic-resonant LN [7] and LN photonic crystal structure [15].

### 2.3. Tunable Second Harmonic Generation

One other fascinating feature of the LN-based device is that it can be tuned via the EO effects. Here in this section, a tunable SHG, where the wavelength of the SH is tuned at a small range with a fixed pump wavelength at 1605 nm, corresponding to the peak wavelength for Δ*n_e_* = 0, is presented. Since the Fano-resonant structure can strongly localize the light field in LN, the EO effect is also enhanced, due to the large field overlay between the localized light and the external electric field. It shows that only under a small voltage excitation, the wavelength of the SH can be tuned and there is only small drop for the conversion efficiency. For the proposed LN-based nanostructure, the in-plane electric field is the dominant component. Consequently, one can estimate the EO effect by only taking into account the largest EO coefficient *r_33_*. Under this assumption, the EO effect can be quantified as follows [18,36]:

(6) $$\Delta n_{e}=\frac{1}{2}n_{e}^{3}r_{33}f_{opt}^{2}E$$

Where *n_e_* is the extraordinary index of lithium niobate which takes the value of 2.136 at 1605 nm, *r_33_* = 30.8 pm/V is the largest component in the EO coefficient tensor of the LN, *f_opt_* is the optical field factor which is 42.42 calculated by Equation 3, and *E* is the external electric field applied via the electrodes. Taking into account the nano electrodes fabrication limit, we assumed a planar electrodes design, with a separation distance d of 20 μm. Consequently, the electric field generated by the external voltage *U* can be approximated by

(7) E=U/d

**Figure 5:**
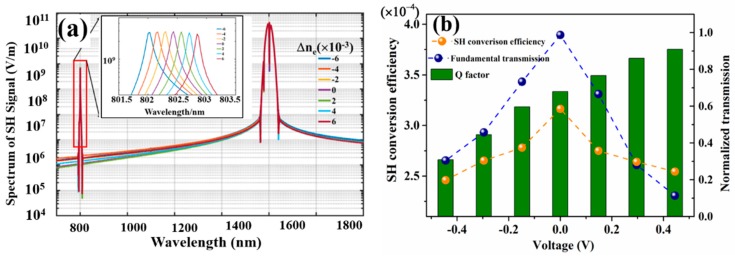
(**a**) Second harmonic signal calculated with different Δ*n_e_* of LN. The inset is a magnified view of SH signal highlighted by red rectangle. (**b**) Quantification of the transmittance (blue spheres), *Q* (olive columns) at the fundamental wavelength of 1605 nm and the conversion efficiency *η* of the SH signal (orange spheres) corresponding to (**a**) where the variable Δ*n_e_* has been transformed into external applied voltage *U* via the EO effect of the LN.

In the 3D-FDTD nonlinear simulations, the EO effect of the LN is taken into account by integrating a Δ*n_e_* for the LN refractive index and the simulated results are shown in Figure 5a. The insets in Figure 5a, show a magnified view of the SH signal corresponding to the different Δ*n_e_*. From these results we can see that under different Δ*n_e_*, both the FW and the SH peak wavelengths have been shifted and further quantification showed that both the FW and the SH peak wavelength shifts quasi-linearly with a linearity of 1.621 nm/V and 0.913 nm/V, respectively. In addition to the peak wavelength shift, there was also transmittance difference under the different Δ*n_e_.* Employing Equation 6 and assuming a planar electrode design, with a separation distance d of 20 μm, one can transform the Δ*n_e_* into the different applied voltages *U*. Figure 5b shows the quantification of transmittance, *Q* of the FW, and the SH conversion efficiency, quantified from the results of Figure 5a, where the Δ*n_e_* has been transformed into the different applied voltage *U*. With the increase of the applied voltage *U*, the peak wavelength of the FW shifts, while the pump light wavelength is fixed at 1605 nm, corresponding to *U* = 0 V. Consequently, the transmittance T decreases from T = 80% for the voltages *U* = 0 V, down to T = 20%, with the applied voltages of *U* = 0. 444 V. Notice that the transmittance decreased with both the increase of the negative and the positive applied voltages. On the other hand, the resonance *Q* of the FW increased with the increase of the applied voltages, where a *Q* of 2540 at *U* = 0.444 V reached the largest value, and a *Q* of 2050, corresponded to the smallest value at *U* = −0.444 V.

As to the SH conversion efficiency, it mainly follows the trend of the transmittance for the FW, which decreased with both the increase of negative and positive applied voltages. The lowest conversion efficiency reached was 2.46 × 10^−4^ corresponding to *U* = −0.444 V, which had only a small drop, compared to the highest conversion efficiency of 3.165 × 10^−4^, corresponding to *U* = 0. With the applied voltage ranging from −0.444 V to 0.444 V, the SH peak wavelength can be tuned from 802 nm, up to almost 803 nm, spanning 1 nm. Whereas, the SH conversion efficiency still kept a high value of 10^−4^, with the largest of 3.165 × 10^−4^.

## 3. Conclusions

In conclusion, combining the all dielectric and wide transparency characteristics of the LN, in a thin-film platform, with a high field confinement design of the Fano resonance, a strong SH signal with a conversion efficiency up to 3.165 × 10^−4^ was achieved. The method of the broken symmetric structure via the asymmetrical distance between the LN bar and the neighboring disks allowed the excitation of a dielectric Fano resonance mode, with high field enhancement in the LN which boosted the nonlinear effects. On the other hand, if one desires an air mode, where the light is confined in the air, then other ways of breaking the structure symmetry, such as creating notches in a cubic nano arrays, might be considered [10]. In addition to the achievement of a high nonlinear conversion efficiency, the electro-optic effect in the LN allows to design a tunable SHG, with external voltages excitations. Further studies might focus on the different electrodes for studying the interaction between the external electric field and the optical field, in order to obtain tunable devices with a large tuning range and high tuning efficiency.

## Figures and Tables

**Table 1:** Comparisons of the SH enhancement or conversion efficiency generated by different materials.

Structure	Material	SH Enhancement	SH Conversion Efficiency	Reference
Broken symmetry Fano metasurfaces	GaAs/AlGaAs	NA	~6 × 10^−5^	[10]
Fano metasurfaces	GaAs	NA	~2 × 10^−5^	[35]
Fano-resonance-based mode-matching hybrid metasurface	ZnS/Au	NA	~5.55 × 10^−8^	[2]
plasmonic-semiconductor hybrid nanosystem	ZnO/Au	~1700	3 × 10^−5^	[8]
Nanobelt-hybrid plasmonic structure	CdS	~1000	2 × 10^−6^	[9]
Gold nanoring resonators filled with lithium niobate	LiNbO_3_/Au	~20	NA	[33]
lithium niobate photonic crystal L3 cavity	LiNbO_3_	NA	6.4 × 10^−9^	[24]
**Nanostructured lithium niobate**	**LiNbO_3_**	**~10^11^**	**3.165 × 10^−4^**	**This paper**
